# First osteological evidence of severed hands in Ancient Egypt

**DOI:** 10.1038/s41598-023-32165-8

**Published:** 2023-03-31

**Authors:** Julia Gresky, Manfred Bietak, Emmanuele Petiti, Christiane Scheffler, Michael Schultz

**Affiliations:** 1 Division of Natural Sciences, German Archaeological Institute, Im Dol 2-6, 14195 Berlin, Germany; 2 Austrian Archaeological Institute, Austrian Academy of Sciences, Dr. Ignaz Seipel-Platz 2, 1010 Vienna, Austria; 3 Human Biology, University Potsdam, Maulbeerallee 12a, 14469 Potsdam, Germany; 4 Institute of Anatomy and Embryology, Göttingen University Medical School, Kreuzbergring 36, 37075 Göttingen, Germany

**Keywords:** Anthropology, Archaeology

## Abstract

For the first time, the severed right hands of 12 individuals have been analysed osteologically. The hands were deposited in three pits within a courtyard in front of the throne room of a 15th Dynasty (c.1640–1530 BC) Hyksos palace at Avaris/Tell el-Dab‘a in north-eastern Egypt. Although this kind of practice is known from tomb or temple inscriptions and reliefs from the New Kingdom onwards, this is the first time that physical evidence has been used to learn more about the procedure and the individuals whose hands were taken. Here, we show that the right hands belonged to at least 12 adults, 11 males, and possibly one female. It is unclear if the hands were taken from dead or living individuals. After removing any attached parts of the forearm, the hands were placed in the ground with wide-splayed fingers, mainly on their palmar sides. The osteological analysis not only supports the archaeological interpretation of this evidence but also adds more detail regarding trophy-taking practices in Ancient Egypt.

## Introduction

The reliability of information must always be questioned, both now and in the past. The more sources available to confirm a story, the more likely it is to be true. Unfortunately, in history and even more so in prehistory, we are limited to a few sources, sometimes only to one. Much information on the lives, habits, and history of the Ancient Egyptians is depicted on temple and tomb walls, as well as recorded on papyri, etc. Like today, information can create certain ideas, exert political influence, and also present facts in a different and not necessarily realistic light. Iconographic and literary sources from Ancient Egypt depict and praise the pharaoh as a victorious military leader. A recurring propagandist motives refers to soldiers presenting the severed right hands of foes to the Pharaoh in order to garner the “gold of honour”^1–9^, a prestigious reward, primarily in the form of a collar of golden beads^4^. Until now, this practice is known only from tomb inscriptions of prominent warriors and from inscriptions and temple reliefs, all dating from the start of the New Kingdom (18th–20th Dynasties) onwards.

**Figure 1:**
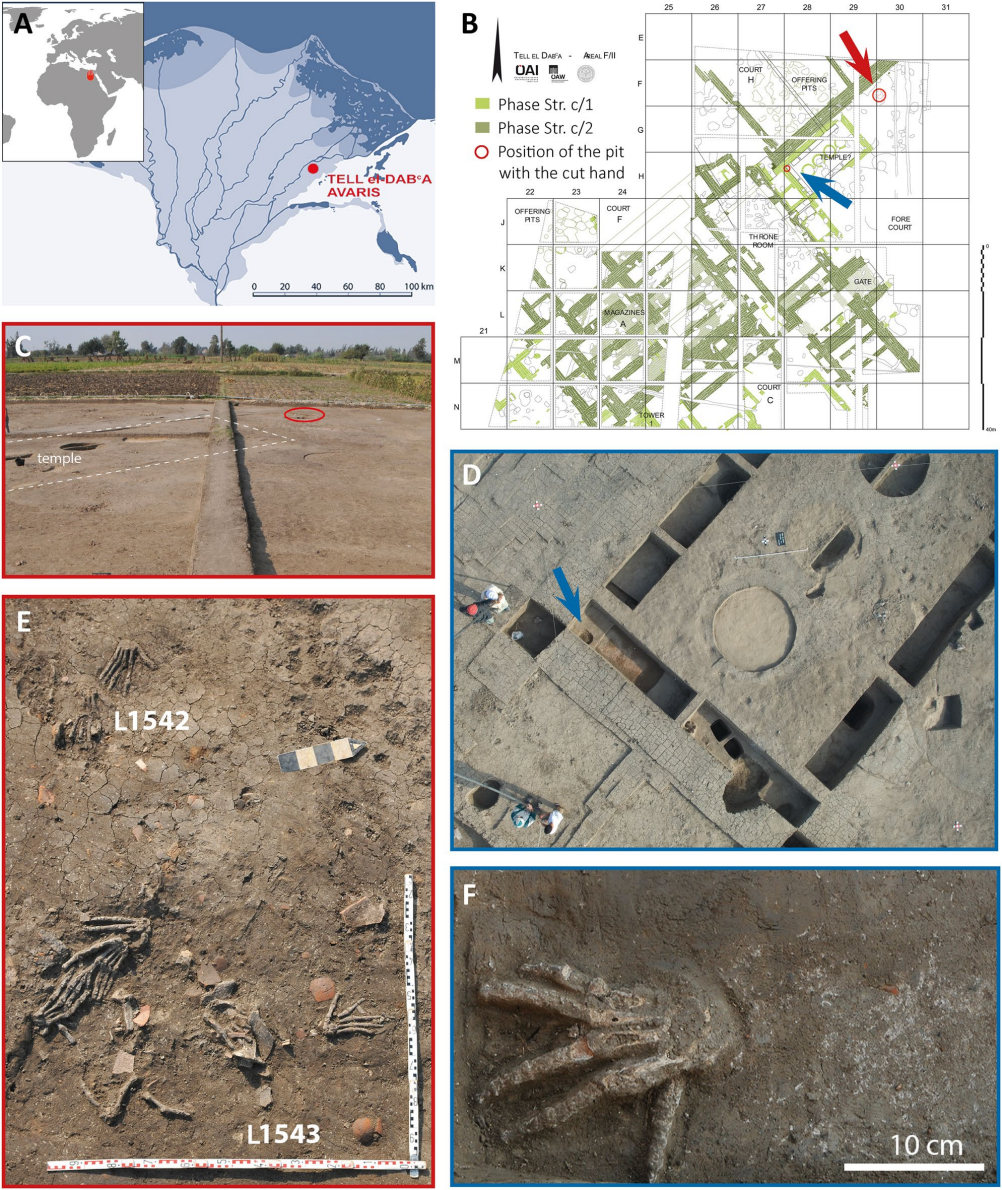
Archaeological evidence of severed hands in Hyksos Period Tell el-Dab‘a, Northern Egypt (images ÖAI, M. Bietak): (**A**) Nile Delta, Northern Egypt (Apple maps) and position of the site Tell el-Dab‘a/Avaris in the Nile Delta. This map has been modified from Fig. 1 (drawn by Nicola Math, modified by Dominik Fill, Austrian Academy of Sciences) in: Manfred Bietak, "Hyksos" in "Hyksos," R. Bagnell, et al. (eds.), Encyclopedia of Ancient History, vol. 6, 1st edition, Malden, MA- Oxford 2013: Wiley-Blackwell, 3356–3362. To publish the map under a CC BY open access license is permitted by the copyright holder Manfred Bietak. (**B**) Northern part of the Hyksos Palace at Tell el-Dab‘a, Phase E1-D3 (after Bietak et al. 2012/13). Red arrow indicating Pit L1542 and 1543, blue arrow indicating Pit L1777. (**C**) Overview of the area of Pit L1542 and 1543 (red circle), the excavation layer closely beneath the modern surface in the agricultural area. (**D**) South wall of a later added broad-room building built against the western enclosure wall of the palace’s forecourt. Pit L 1777 in front of the throne room (indicated by the arrow). (**E**) Overview of the 11 right hands in the Pits L1542 and L1543. (**F**) Single right hand on its palmar surface with wide-splayed fingers.

The bioarchaeological analysis of human hands, found in 2011 at Tell el-Dab‘a (ancient Avaris) in the eastern Nile delta region^5,10–13^ (Fig. 1A), provides a third source of evidence (additional to iconographic^1–4,6–9^ and archaeological^5,10^), offering unique insights and, to date, unknown details of this practice. Taphonomic and biological analyses carried out on the bones reveal information regarding the act of mutilation and preparation of these body parts, as well as about the individuals to whom they originally belonged.

From a wider transdisciplinary scenario, the results presented in this article address questions about the embodiment of violence in the context of war, and, specifically, trophy taking as a structured language of dominance.

## Results

### Materials and archaeological context

In the forecourt of a Hyksos Period Middle Bronze Age-style palace (c. 1640–1530 BC)^11–13^, three pits with severed hands were found^5,7,8,10^ (Fig. 1). The palace was built on top of a similar 14^th^ Dynasty palace and had a longer lifespan, covering the major part of the Hyksos Period. One of its main occupants seems to have been the Hyksos Khayan (c. 1700 BC–1580 BC), whose numerous seal impressions were found in offering pits belonging to its earlier phase^11–15^. Judging from its later offering pits and the filling of a well from the later phase, the palace may have been used until the late Hyksos Period but may have lost its purpose when a new palatial compound was built further north^15^ in the late Hyksos Period.

The smallest of the three pits, Pit L1777, in front of the throne room, contained a single fully articulated hand sealed beneath the south wall of a later added broad-room building, most probably a temple built against the western enclosure wall of the palace’s forecourt. The pit seems to have been dug into the temple’s open foundation trenches because it cut the continuous foundation layer of loam-mortar at the base of the trench (Fig. 1B,D,F). It can, therefore, be dated in relative terms between the early and the late phase of the palace. Two more pits were discovered c. 7 m north-east of the broad-room building near the western enclosure wall, just below the modern agricultural fields (Fig. 1B,C,E). These two pits were aligned to the enclosure wall of the palace’s forecourt and no ceramic material later than the Hyksos Period was found inside them. Pit L1542 contained the remains of three and L1543 the remains of eight articulated hands, thus the right hands of 12 individuals. In both pits there were also disarticulated fingers, their attribution will be discussed below.

### Bioarchaeological evidence

#### Number of individuals

The superficial position of the hands in the ground led to heavy erosion, flaking and cracking of the bone tissue. The high humidity and soil composition make them soft and brittle and very difficult to excavate (see chapter limitations). Referring only to the quantitative state of preservation, the hands will be grouped in three classes. Complete: at least 75% of the hand bones are present and articulated; Almost complete: 75–50% are present and articulated; Single digits: when a complete digit (including metacarpal bone, proximal to distal phalanges) or parts of them are present.

Anatomical markers identify all hands and single phalanges from the three pits as being from right hands (for more detail, see Supplementary Information). L1777 comprised one complete right hand, while L1542 comprised two complete right hands together with an incomplete single second right digit belonging to a third person. Pit L1543 contained eight complete and almost complete right hands and eleven additional phalanges that partly belong together, leaving six additional complete or incomplete digits. Therefore, the evidence suggests a minimum of 12 right hands, hence at least 12 individuals, from the three pits in front of the throne room and along its wall (L1543: 8 hands, L1542: 3 hands, L1777: 1 hand). Assuming that the six single digits each represent an extra hand, that would result in a maximum of 18 right hands in the three pits.

#### Deposition

**Figure 2:**
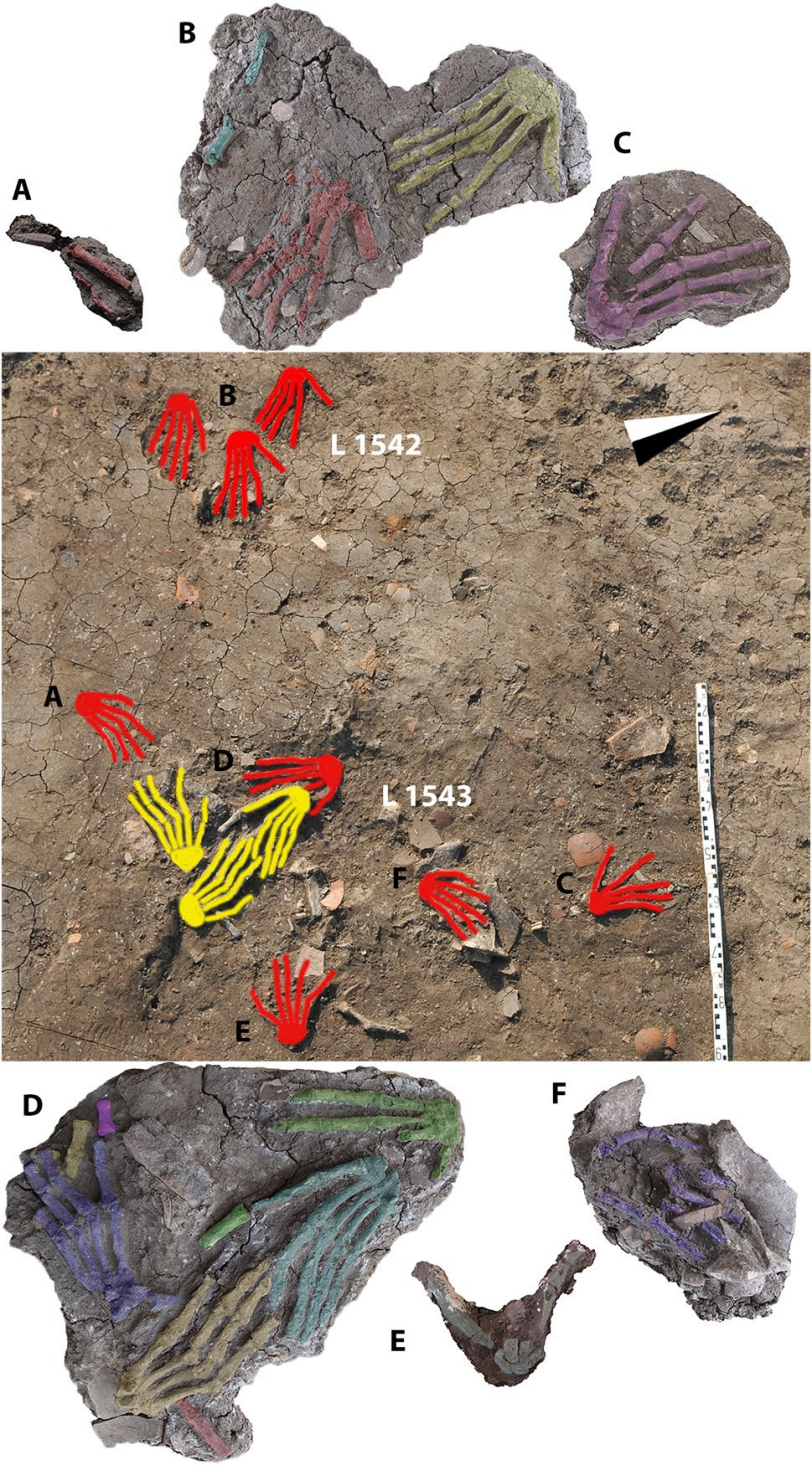
Anthropological reconstruction of the finding and details of the right hands of Pits L1542, L1543 (reconstruction and images of single hands, J. Gresky): reconstruction of the complete hands in the Pits L1542 (left upper corner) and L1543 (lower half of the picture). Yellow hands are placed on their dorsal surface whereas the red ones are placed on their palmar. The missing elements are reconstructed. (**A**,**C**–**F**) eight right hands of Pit L1543, together with single phalanges which could either represent additional hands or might belong to the present hands. The preserved bones are coloured. (**B**) Three right hands on their palmar surface in Pit L1542. The preserved bones are colour.

Of eleven complete right hands (see Table 1 in supplementary information), eight are placed on their palmar surface and three on their dorsal surface (Fig. 2). The fingers of six hands were splayed wide; the fingers of four hands were lying close together; and in one hand, the position of the fingers could not be determined. There is no correlation between the position of the fingers and the placement on the dorsal or palmar side. Because the single phalanges might have been moved after the placement of the hands, the informative value of their position is low. The metacarpophalangeal joints of the two intact hands found in L1542 were hyper-extended, so were the hands in L1543-7 and L1543-8. L1543-8 also showed a misplacement of the first digit, which was hyper-abducted (98°), exaggerating the maximum value of 45° between the first and second digits. L1777 had a displaced first digit, with the proximal metacarpal and the radial carpals positioned below the rest of the hand.

Following the deposition of the hands, some post-mortem displacements might have occurred: the first digit of one complete hand (L1543-8) was either disarticulated before or after the hand was placed in the ground. Six single fingers/phalanges were scattered between the other, complete and almost complete, hands. Either these elements were moved from the intact hands or they represent the remnants of additional hands that were displaced by rodent activity or originated from other such pits that were disturbed by the later interments of hands. Post-mortem displacement of these elements would have required leaving the hands in the pit open for a period of time, at least until the soft tissue decomposed and elements could be moved. However, there are no animal gnawing marks on the bones. Therefore, it seems unlikely that the pit was left open for an extended period of time.

The position of the hands, mainly on their palmar surfaces with splayed fingers, might have been caused either by taphonomic reasons, or it might have been due to their deliberate placement. In the first case, when the hands would have been thrown into the pits, soil pressure would have flattened them, by pressing the arch of the hand into the ground, possibly leading to splaying of the fingers and hyperextension of the metacarpophalangeal joints^16^. If we assume a deliberate placement, this arrangement could have been done to make the hands look more impressive, possibly larger, and to better match the prototype of a hand. Yet, there is no pattern in the placement of the hands; some were single, some lay on top others in a smaller group.

#### Severing and preparation

Only six hands have preserved proximal row carpal bones, and none exhibit cut marks or any evidence of soft tissue removal. Because no fragments of lower arm bones were attached or found in the pit, it implies that these hands were precisely severed from the lower arm. One technique of severing hands is to cut the joint capsule and open it by intersecting the tendons spanning the wrist joint^17^. If done correctly, there are no cut marks on the bones. If done unprofessionally, however, cut marks are to be expected. Mutilating people without regard to their survival is often done by severing the arm at any anatomical position. This method is faster and easier, but it leaves a section of the lower arm attached to the hand. If this was the case with these hands, the people offering them, or those in charge of the ceremony, cared enough about their proper presentation to detach parts of the lower arm.

Two main distinctions can be made regarding the procedure of hand detachment: collecting them from the recently deceased or mutilating living people. In both cases, the hands must have been soft and flexible when they were placed into the pit. That is, either before rigor mortis sets in or after it has resolved. Rigor mortis of the hands commonly begins 6–8 h after death (there are different times for different body parts). This means that living victims were mutilated during or shortly before the ceremony. It seems, however, much more likely that the hands were placed after rigor mortis ended, between 24 and 48 h after death. This indicates that the hands were collected and kept for a period of time before being placed in the pit. The hands were buried while they were still intact, at least with the tendons and ligaments holding the skeletal elements in their original place and remaining supple enough to flex passively under appropriate stress. This capacity is affected by surrounding environmental factors like humidity and temperature.

#### Biological profile of the hands

The closed epiphyseal lines indicate that all the specimens belonged to adult individuals older than 14–21 years^18^. The absence of even incipient bone changes owing to age-related degenerative processes, e.g., DJD and osteoporosis, rules out individuals reaching the old adult age class.

The large size and robustness of the hands point to the male rather than female sex of the individuals. However, the size of hands varies between males and females. Because genetic analyses to determine the sex could not be applied due to the very poor preservation of the bones (see “Methods”), an estimation of the 2D:4D ratio was employed to determine the sex of the individuals^19–21^. The typical male 2D:4D ratio is that the fourth digit is longer than the second. The proportion of these two fingers is different in males and females due to prenatal exposure to androgen^22^. The measurements of the phalanges of all the hands show the fourth digit to be longer than the second, indicating male sex (see Supplementary Information). The sole possible exception to the 2D:4D ratio is the L1543-2 hand, which macroscopically appears smaller than the others. This hand’s 2D:4D ratio suggests a female (SDS of 2D:4D ration is beyond the normal range). However, in this case the phalanges of the second and fourth finger were partially incomplete, thus measurements could only be estimated.

### Interpretation and contextualisation

The bioarchaeological evidence from Tell el-Dab‘a addresses the question, crucial to its interpretation, of whether the mutilation occurred as a form of punishment or as an accounting and reward system following military victories.

Thus far, the severing of hands as a punishment is not attested in Egyptian texts. Nevertheless, the removal of right hands is mentioned by papyrus Salt 124, l, 7, from the 20th Dynasty^7,23,24^. This deals with the act of plundering Sety II-Merenptah’s royal tomb. During the plunder, the pharaoh’s hand was removed, apparently by the tomb robbers, perhaps to obtain quickly the rings from the fingers. Indeed, the right hand of the mummy of Sety II is missing, and hand removal is also attested from other royal mummies, most likely for this reason^25,26^.

The pits containing the hands were located in the palace’s forecourt, in front of the throne room. Their position points to the widespread visibility conferred by the practice that generated the deposits as part of a public ceremony. The later attested ‘Window of Appearances,’ through which New Kingdom kings offered the ‘gold of honour,’ may have already existed in this palace^27^.

The absence of the distal parts of the lower arm and the lack of cut marks indicate the hands underwent a careful pre-depositional preparation phase, aimed at removing all elements deemed to be unrelated to the anatomy of the hand. At the moment of their deposition in the pits, the hands might have been arranged, predominantly on their palmar faces (n = 8/11), in most cases with the fingers spread out (n = 6/10). Assuming an intentional positioning, this appears aimed at facilitating the identification of the body part in the pit as a hand, thus conferring on it a defined semiotic function. In the iconicity of this position^28^, each hand represents an individual: *pars pro toto*.

It appears that all the hands belonged to adult individuals who may not have reached late adulthood. The results also suggest that all the individuals were likely males, except for a possible female. If we refer to the male warrior hypothesis, long debated in behavioural sciences^29^, this data may support the scenario presented above, because the severed hands offered in the “gold of honour” ceremony belonged to foes, generally male individuals of fighting age, killed in battle. At the same time, the presence of a female individual advocates for a less gender-rigid approach to the reconstruction of this procedure. Throughout history, women have played various roles in military societies. Women and warfare did not exist in separate worlds. On the contrary, they were inextricably linked to the political, social and religious spheres^30,31^. Consequently, we cannot exclude that the specific hand attested at Tell el-Dab‘a belonged to a woman.

Although the hands cannot be attributed to a specific ethnic or cultural group, the custom of severing the right hands of foes appears to have been introduced to Egypt by the Hyksos^2,5^, approximately 50–80 years earlier than the inscriptional and pictorial evidence. The Egyptians adopted this custom at the latest in King Ahmose’s reign, as shown by a relief of a pile of hands at his temple in Abydos^14^ and in the autobiographies of Ahmose, son of Ibana^32,33^, and Ahmose, son of Pennekhbet in El-Kab^32,34^. Ahmose was the one who conquered Avaris and defeated the Hyksos, and thus was likely familiar with this practice. Early 18th Dynasty tomb inscriptions and temple reliefs from the 18th to the 20th Dynasties consistently depict hand counts on the battlefield following major battles^1–4,6–9^.

**Figure 3:**
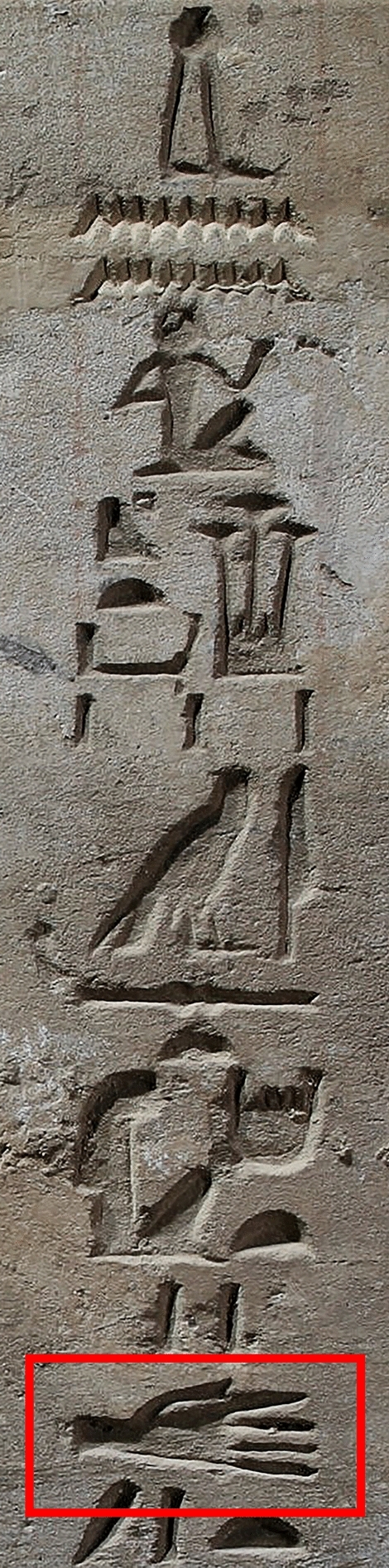
Iconographic evidence of severed hands: inscription in the tomb of Ahmose at El-Kab depicting a very realistic representation of an outstretched palm, showing five spread fingers (Courtesy of William Vivian Davies, Oxford). Publishing this figure under a CC BY open access license is permitted by the copyright holder Vivian Davies.

The idea that the custom of severing enemy hands originated in the Near East may be supported linguistically^9^. During the early 18th Dynasty, a specific new hieroglyph appears with the first inscriptional mention of the cutting of hands in warfare in the inscription of Ahmose, son of Ibana. It is not the typical logogram-sign for hand *d.t* in side view (Gardiner list, D46) but a very realistic representation of an outstretched palm, showing five spread fingers (Fig. 3). This new pictogram may signal a new word instead *d.t* ‘hand’—which now also refers to ‘severed hand.’ In Egyptian inscriptions beginning with the 19th Dynasty (Merenptah), a Semitic loanword *kp* כף ‘hand’ ‘palm’ is introduced to refer specifically to ‘severed hands’^9,35^. In the hieroglyphic inscriptions of the 20th Dynasty kings, the new word only refers to ‘severed hands’^35^. It seems possible that this semitic loanword entered already in the 18th Dynasty into military language.

The outstretched hand symbol is a compelling parallel to the bioarchaeological evidence of this paper. Although it is beyond the scope of this article to discuss the possible origins of this practice, it should be noted that corporal mutilation of enemies has been known in Egypt since the time of King Narmer in the Early Dynastic period, but the severing of hands appears in Egyptian records only after Hyksos rule. Seals with rows of possible severed hands and heads, together with animal heads, also appear in Middle Bronze Age Syrian glyptic art^36^.

Although bioarchaeological literature offers abundant evidence of trophy-taking practices from all over the world^37,38^, the case presented here documents a marked visual component, as the body parts, i.e., severed hands, were prepared and arranged for presentation in a public ceremony in the pharaoh's palace. In this politically structured frame, the severed hands served as symbolic currency for status acquisition, within a system of values celebrating warfare and dominance, as argued by other bioarchaeological studies in geographically diverse contexts^39^.

Iconographic sources from several archaeological sites in Egypt deliver a large corpus of evidence for the practice of body dismemberment and mutilation, particularly related to war contexts^40,41^. The representation of piles of severed heads, ears and genitals^42^ follows codified and publicly recognisable ethics of violence^43^ as it ultimately conveys a message of political stability: the pharaoh, representation and personification of the gods, maintains the universal order by defeating the forces of chaos, personified by the enemies^44^. The public enactment was the necessary step to deliver this message.

Here, the act of dominance is conveyed by maiming defeated enemies, depriving them of their right hands, hampering their capacity to carry out future attacks and essential daily-life activities. The likelihood that hands were taken from captives is low, since this would limit their potential as future slaves^45^. Because corporeal integrity was vital for survival in the Ancient Egyptian view of the afterlife^46^, the victim’s impairment adds a deeper dimension to this act of dominance.

## Conclusions

The location, treatment, and possibly the positioning of the severed hands argues against the hypothesis of law-enforcing punishment as the motivation for these acts. When contextualised in a transdisciplinary approach to the archaeological and historic sources, the bioarchaeological evidence presented here suggests that the severed hands were offered as trophies as part of a public event that took place in the palace. They belonged to at least eleven males and possibly one female, which may indicate that women and warfare were not worlds apart. To the best of the authors’ knowledge, the results put forward in this paper provide the first direct bioarchaeological evidence for the ‘gold of honour’ ceremony performed in front of the king’s palace and contribute significantly to the debate over the reconstruction of this ceremony.

## Methods

### Osteology

At the excavation site, the hands were hardened with acetone-soluble glue and recovered *en bloc* in a plaster cast. In the lab, the very fragile bones remained in the plaster casts; only the surfaces were carefully cleaned of adhering sand. Unfortunately, a thin layer of calcareous deposits has covered some surfaces. This could not be removed since proper cleaning would have resulted in surface destruction.

The bones were examined in detail using a magnifying glass. The surfaces of all bones, but particularly the proximal carpal bones, were inspected for cut marks.

The hands were measured in situ using a sliding calliper (maximum length of phalanges, distances between the fingers; maximum width and length of the hands).

The minimum number of individuals (MNI) was calculated by excluding skeletal elements that could belong to other hands.

Age estimation was carried out on the epiphyseal closure of the metacarpals and phalanges^18^. Additionally, when the bones were broken, the state of osteoporosis of the compact and spongy bones was estimated, and the joint surfaces were assessed for degenerative changes.

### Osteometry

The 2D:4D ratio is sex-based, with the fourth digit typically being longer than the second in males^19,20^. In living people, digit length is “measured on the ventral surface from the basal crease to the tip of the digit”^20^. By adapting measurements from living people to archaeological skeletons, we proposed that the ratio of the length of the single phalanges of the fourth and second digits should differ as well.

The measurements of all complete phalanges were taken (Table 2 in supplementary information). The 2D:4D ratio for each phalanx of the second and fourth digit was determined, and the individual mean and the standard deviation score (SDS) of the 2D:4D ratio were calculated (Table 3 in supplementary information).

The SDS is the individual value subtracted by the mean of a reference divided by the standard deviation of a reference. In our case, we calculated the ratio from each existing pair of phalanges of the second and fourth finger, and calculated the mean (individual value) for each individual. The sum of individuals is our reference. As a result, we have standardised dimensionless values (SDS) that enable us to compare individuals.

Long bones are always distributed normally in a population^47,48^. Measurements between the ± 1.28 SDS range are significant (Fig. 1 suppl).

### Limitations

The excavation of human remains in this particular region of Egypt is very difficult due to the high humidity of the ground and the soil chemistry^49^. The humid and dark soil affects the bones, which stains them and giving them a similar colour to the soil. It also makes them soft and brittle. As a result, they are extremely difficult to detect and to fully excavate without causing damage. Small body parts, like hands, are especially vulnerable to being overlooked, and by the time they become visible, parts of them may have already been lost.

The 2D:4D ratio for sex estimation applies to living people. We transferred the method to archaeological skeletons, which is not exactly comparable to the data obtained from living people. However, the measurements taken from the bones should be even more exact due to the missing tissue, which can confound the measurements of finger lengths. There is no reason to assume that the influence of soft tissue on fingers is sex specific.

## Supplementary Information

Supplementary Information.
