# Supplementary material for: First osteological evidence of severed hands in Ancient Egypt
Source: Sci Rep. 2023 Mar 31;13:5239. doi: 10.1038/s41598-023-32165-8 (PMC10066219; doi:10.1038/s41598-023-32165-8)
Supplement: Supplementary file 1 — Supplementary Information. [file 41598_2023_32165_MOESM1_ESM.pdf]

Supplementary Information: Gresky et al. 2022 Severed Hands

A. Detailed descriptions of the single hands and osteometric results

feature no.	hand no.	site	lying on	position	preservation	max. width hand in mm[1]	max. width metacarpus in mm[2]
1542	**hand 1**	right	palmar	fingers spread	complete hand		69
1542	**hand 2**	right	palmar	fingers spread	complete hand		approx. 65
1542	**hand 3**	right	radial	indeterminable	single finger		
1543	**hand 1**	right	palmar	fingers spread	complete hand		63
1543	**hand 2**	right	palmar	fingers closed	disturbed hand	115	62
1543	**hand 3**	right	palmar	fingers spread	disturbed hand	175	
1543	**hand 4**	right	palmar	indeterminable	disturbed hand		
1543	**hand 5**	right	palmar	fingers closed	disturbed hand		
1543	**hand 6**	right	dorsal	fingers closed	complete hand	140	67
1543	**hand 7**	right	dorsal	fingers closed	disturbed hand		77
1543	**hand 8**	right	dorsal	fingers spread	complete hand		68
1543	**hand a**	indet.	dorsal	indeterminable	single finger		
1543	**hand b**	indet.	palmar	indeterminable	single phalanx		
1543	**hand c**	indet.	palmar	indeterminable	single phalanx		
1543	**hand d**	right	palmar	indeterminable	single finger		
1543	**hand e**	right	radial	indeterminable	single finger		
1543	**hand f**	right	palmar	indeterminable	single finger		
1777	**hand 1**	right	palmar	fingers spread	complete hand	190	69

Tab. 1 Single right hands and phalanges from the three contexts L1542, L1543, and L1777

[1] maximal hand width in mm: the distance between the distal phalanges of the first and fifth digit.

[2] maximal metacarpus widths in mm: distance between the heads of the second and fifth metacarpal bones.

length in mm	metacarpals					proximal phalanges					middle phalanges				distal phalanges				
Digit no.	**1**	**2**	**3**	**4**	**5**	**1**	**2**	**3**	**4**	**5**	**2**	**3**	**4**	**5**	**1**	**2**	**3**	**4**	**5**
1542-1	48	66	66	58	56		36	48	45	36	30	35	35	25					
1542-2		67	70				39		45	44		27	31	26			20		21
1542-3							41												
1543-1	43		56	54	50	39	36	40	40	32	23	27	27	20	14		17	19	
1543-2	45				52	31	42	43	40	34			27	22	20				13
1543-3					49	34				29					23				22
1543-4									43	29			29	21					18
1543-5				61	56				47	37			33	22					19
1543-6	48	66	66	60	60	37	42	50	46	38	25	29	29	24	24	17	21	20	18
1543-7		72	70	67	58		45	50	46	35	28	35	32	23			21	21	
1543-8		66	66	62			42	48	42	33	27	30	29						
1543-a						34													
1543-b						34													
1543-c								51											
1543-d	48																		
1543-e		71					41				24								
1543-f						33													
1777		64	60	51		29	39	42	39		23	29	28	20	23	17	18	19	17

Tab. 2 Maximal lengths in mm of the metacarpals and phalanges of the hands

Empty boxes: no measurements could be taken.
Blue numbers: approximate measurements due to incomplete bone endings.

hand no.	individual mean D2:D4	SDS	max. width of the hand in mm	max. width of the hand SDS	max. width of the metacarpus in mm	max. width of the metacarpus SDS
1542-1	0.83	-1.268			69	0.321
1542-2	0.87	-0.688			65	-0.536
1543-1	0.88	-0.543			63	-0.965
1543-6	0.88	-0.543	140	-0.218	67	-0.107
1777	0.92	0.036	190	1.091	69	0.321
1543-7	0.94	0.326			77	2.039
1543-8	0.97	0.760			68	0.107
1543-2	1.05	1.920	115	-0.872	62	-1.180
mean	0.917		148.333		67.5	
SD	0.069		38.188		4.659	

Tab. 3 Standard deviation score (SDS) and mean of the 2D:4D ratio, hand maximal width and metacarpus maximal width in mm.

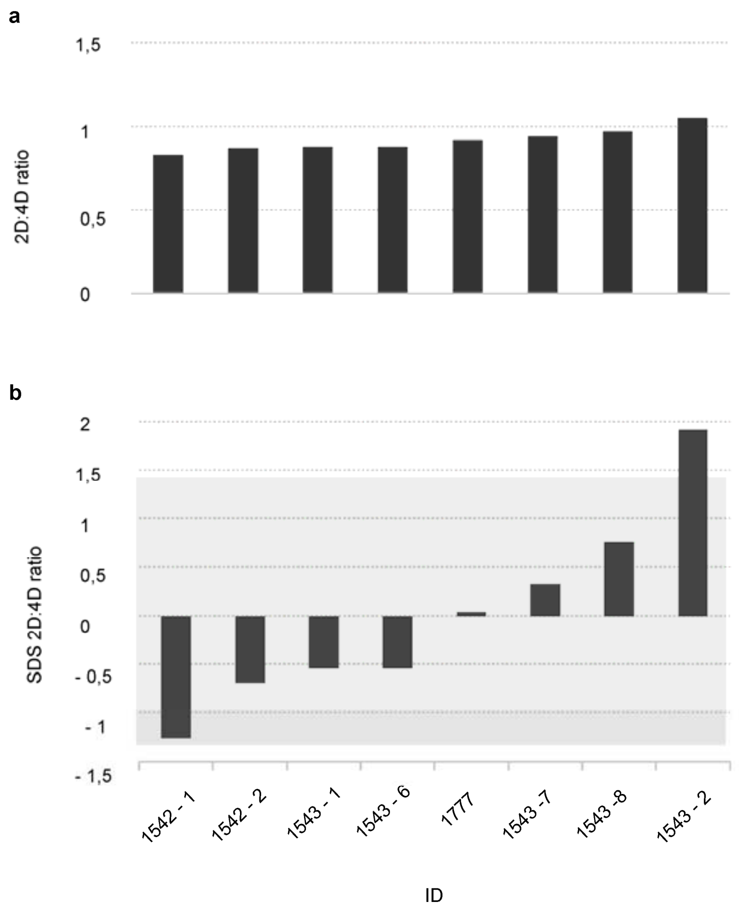

Fig. 1 Graph of table 3: D2:D4 Ratio (mean and standard deviation score (SDS)) of all second and fourth digit phalanges measured for each individual (ID).

a: mean of each individual,
b: SDS value of each individual, blue box indicates the normal range of +/- 1.28 SDS

Supplementary Information: Gresky et al. 2022 Severed Hands

B. Documentation of the hands

In-lab pictures colour legend: each hand is coloured to show the bones present for analysis.
Schemes colour legend: dark grey: bone surface preserved; light grey: bone surface eroded but bone was present; white: bone not present for analysis

L1542

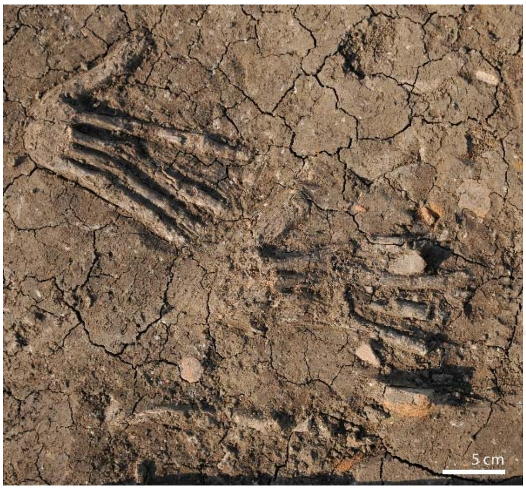

In situ

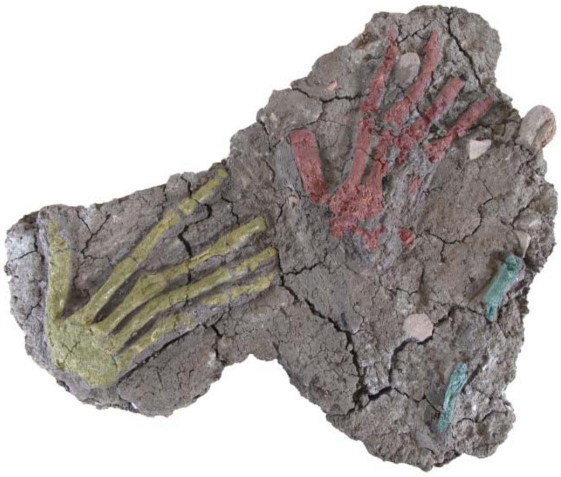

In lab

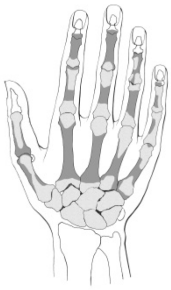

Hand L1542-1 (yellow): a single right hand placed on its palmar surface. Preservation is very poor; the distal and proximal ends of the single bones are destroyed. The hand ends with the carpals; no evidence of lower arm bones. The carpals cannot be assessed for cut marks due to their poor preservation. The fingers are spread, the first digit 38° abducted. The hand is hyper-extended in the metacarpophalangeal joints. The phalangeal joints are slightly flexed to straight. The largest distance is between the second and third phalanges. Ulnar deviation, particularly of the third to fifth finger.

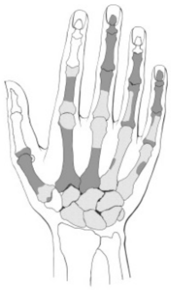

Hand L1542-2 (red): a single right hand placed on its palmar surface. Preservation is very poor; the distal and proximal ends of the single bones are destroyed. The hand ends with the carpals; no evidence of lower arm bones. The carpals cannot be assessed for cut marks due to their poor preservation. The fingers are spread, and the first digit is 30° abducted. The hand is hyper-extended in the metacarpophalangeal joints. The phalangeal joints are slightly flexed to straight. Slight ulnar deviation of all fingers.

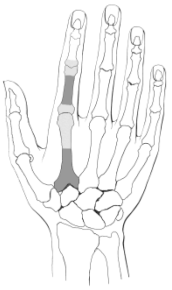

Hand L1542-3 (blue): a single right second finger placed on its radial surface, possibly discarded and belonging to another hand. The distal part of the metacarpal bone is missing. The phalanx next to it is too far away to be the proximal, and for being the middle it is too close to the metacarpal. Based on its length, it appears to represent a proximal phalanx.

Supplementary Information: Gresky et al. 2022 Severed Hands

L1543

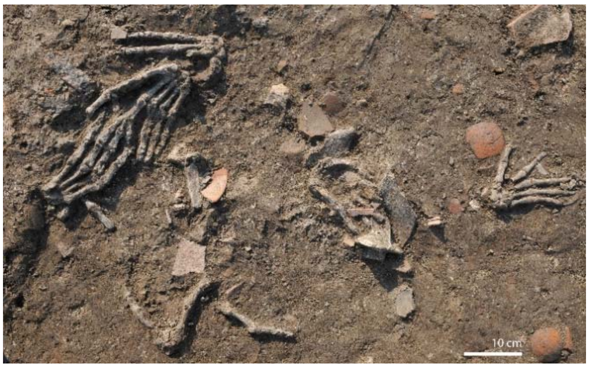

L1543-1

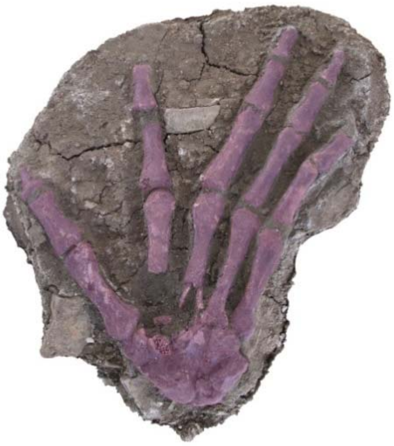

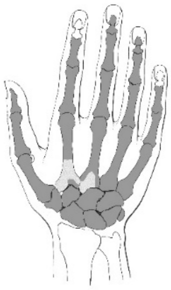

A single right hand placed on its palmar surface. Preservation is good; the surfaces are well preserved despite the adhering glue. The hand ends with the carpals; no evidence of lower arm bones. The carpals cannot be assessed for cut marks due to their poor preservation. The fingers are spread, and the first digit 36° abducted. The largest distance is between the second and third phalanges. Ulnar deviation, particularly of the third to fifth fingers.

L1543-2

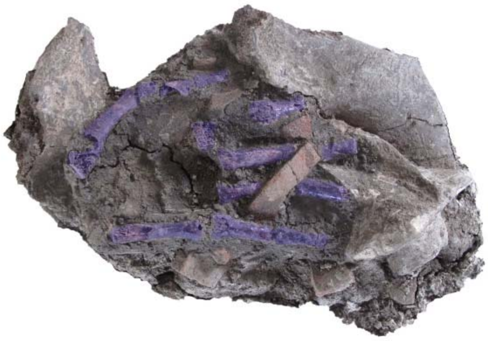

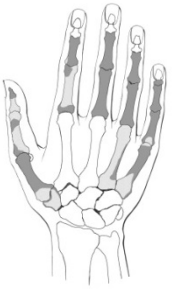

A single right hand placed on its palmar surface between two big animal bones, lying between ceramic sherds. The preservation of the hand is poor. Because the carpals and metacarpals have not been preserved, they cannot be assessed for cut marks. The hand is small, with fingers closed rather than spread.

L1543-3

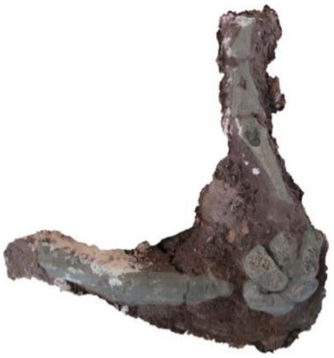

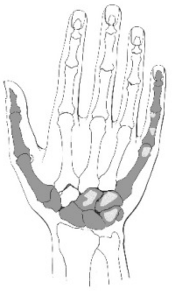

A single right hand placed on its palmar surface. Preservation is poor; the carpals were damaged post-mortem on their dorsal surface. The second to third digits are missing due to post-mortem destruction. The hand ends with the carpals; no evidence of lower arm bones. The carpals cannot be assessed for cut marks due to their poor preservation. The fingers are spread, lying stretched and flat.

L1543-4

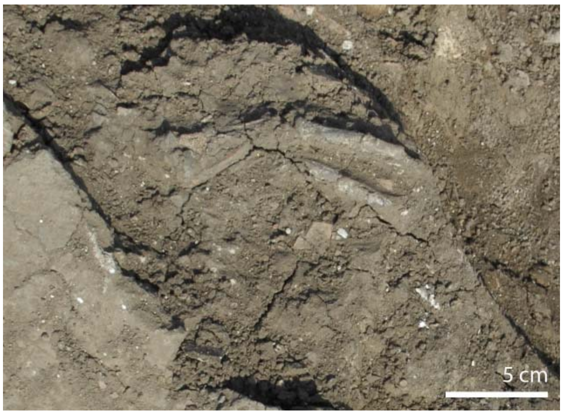

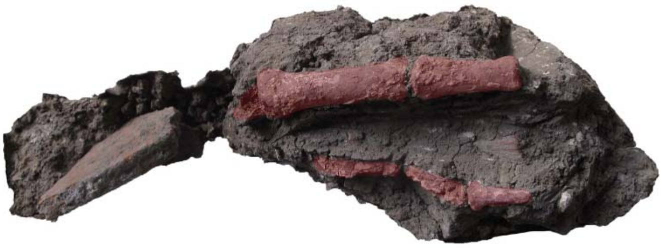

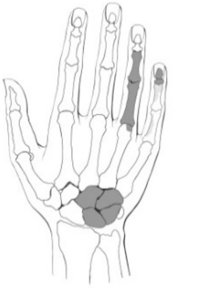

The proximal and middle phalanges of the fourth and fifth fingers of a right hand are placed on the palmar side. The distal part of the fourth metacarpal, as well as the capitate, lunate and triquetral bone, are present. These bones were not hardened with glue and were, thus, falling apart. The hand ends with the carpals; no evidence of lower arm bones. No evidence for cut marks on the proximal carpals. The fingers are spread, lying stretched and flat.

Supplementary Information: Gresky et al. 2022 Severed Hands

L1543-5 – 8

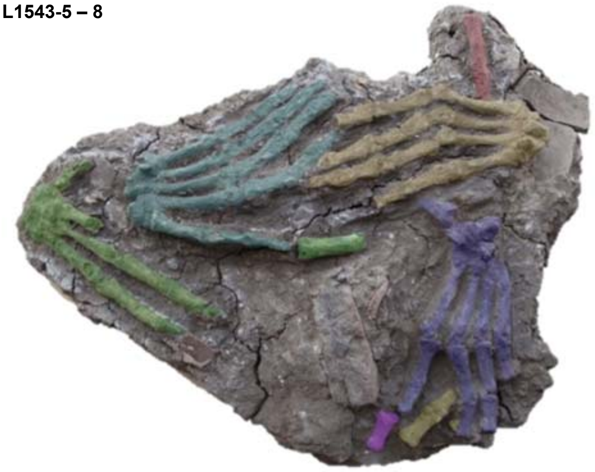

Four almost complete hands in one cast, together with some single phalanges that could possibly belong to the hands.
The hands must have been soft and flexible when they were placed into the pit, as hands L1543-7 (yellow) and L1543-8 (violet) are hyper-extended in the metacarpophalangeal joints. The hands L1543-5 (green) and L1543-6 (turquoise) are flat, with the fingers only deviating to ulnar. These two hands must have been covered by soil soon after being deposited as the first digit of hand L1543-6 (turquoise) is still in place like it would be in an intact hand and has not moved due to decomposition.
The hands were placed in the following order: Hand L1543-5 (green) was placed first, possibly together with hand L1543-8 (violet). Hand L1543-6 (turquoise) was placed on top of hand L1543-5 (green), followed by hand L1543-7 (yellow).
Single phalanges are distributed in different areas of the cast. By their robust appearance, the single proximal phalanx L1543-c (green), as well as the proximal phalanx of the first digit L1543-b (pink), could belong to hand L1543-5 (green). However, they would have been too protracted. The proximal and distal phalanges of the first digit L1543-a (yellow) seem to be in situ; they belong to another hand rather than to L1543-5 (green). The first metacarpal and proximal phalanx (red) next to hand L1543-7 (yellow) may belong to this hand, although it has been too extensively moved, pointing rather to another hand.

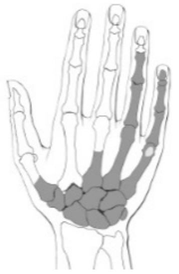

Hand L1543-5 (green): a single right hand, the only one in this group placed on its palmar surface. Preservation is poor, the surfaces covered by a hardened layer of calcareous deposits. Most of the first to third digits are missing. The hand ends with the carpals; no evidence of lower arm bones. The carpals do not show cut marks. The fingers are closed rather than spread. The proximal part of the first digit is slightly abducted.

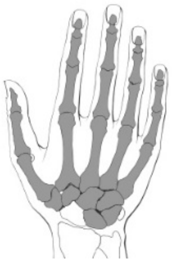

Hand L1543-6 (turquoise): a single right hand placed on its dorsal surface in the same direction as hand L1543-5 (green). Preservation is very poor; the proximal row of the carpals is missing, most probably post-mortem. No evidence of lower arm bones. The fingers are closed rather than spread. The middle and distal phalanx of the second digit show a 20° ulnar deviation, the other digits are relatively straight. The second digit was possibly moved when hand L1543-7 (yellow) was deposited.

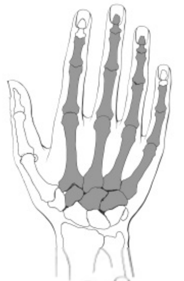

Hand L1543-7 (yellow): a single right hand placed on its dorsal side opposite L1543-6 (turquoise), the fingers touching each other. Preservation is very poor; the proximal row of the carpals is missing, most probably post-mortem. No evidence of lower arm bones. The fingers are closed rather than spread. The second to fourth digits are hyper-extended in the metacarpophalangeal joints; the fifth digit is relatively straight. There is a first digit (red) next to the hand, but it is in the opposite position to what one expects for a digit in situ.

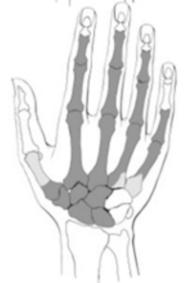

Hand L1543-8 (violet): a single right hand placed on its dorsal side vertical to hand L1543-7 (yellow), the first digit being placed below the proximal phalanges of hand L1543-7. Preservation is very poor. The hand ends with the carpals; no evidence of lower arm bones. No evidence of cut marks on the proximal carpals. The fingers are closed rather than spread. The second to fifth digits are hyper-extended in the metacarpophalangeal joints; the angle of the fifth digit measures approximately 35°. Ulnar deviation of all phalanges is visible. The first digit is hyper-abducted by 98°, exaggerating the maximum value of 45° between the first and second digits. It was either disarticulated before the placement of the hands or later, and discarded.

Hand L1543- single phalanges (a: yellow, b: pink, c: green, d: red)

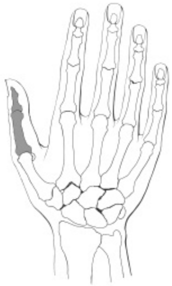

Phalanges L1543-a: proximal (length 34mm) and distal phalanges of the first digit (yellow) lying on their dorsal surface and reaching below the fingers of hand L1543-8 (violet).

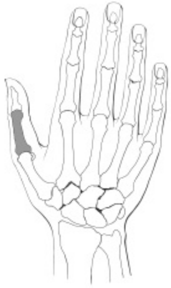

Phalanx L1543-b: a single proximal phalanx (length 34mm) of the first digit lying on its palmar side (pink).

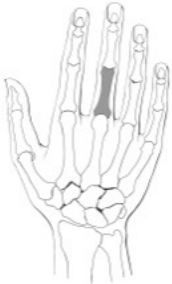

Phalanx L1543-c: a single proximal phalanx (length 51mm) possibly of the second to fourth digit (green), lying on its palmar surface, might belong to hand L1543-5 (green) and shifted distally.

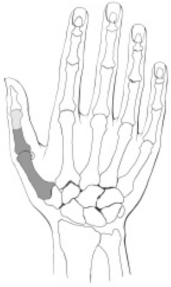

Phalanges L1543-d: a single first right digit (red) of metacarpal bone (length 48 mm) and a proximal phalanx lying on the palmar surface. It is placed below the metacarpals of hand L1543-7 (yellow), but probably does not belong to it as it is lying vertical and shifted to the metacarpals. It was either disarticulated before the placement of the hands or later, and discarded. It definitely could not have fallen into this position by natural decomposing processes.

L1543-e

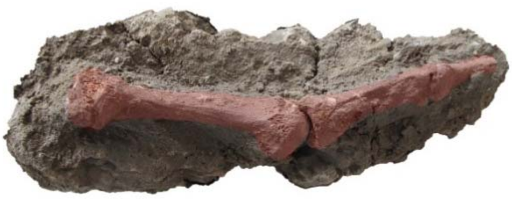

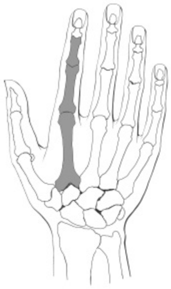

A single second right finger lying on its radial surface. The metacarpophalangeal joint is hyper-extended by 28°. The proximal interphalangeal joint is straight.

Supplementary Information: Gresky et al. 2022 Severed Hands

L1543-f

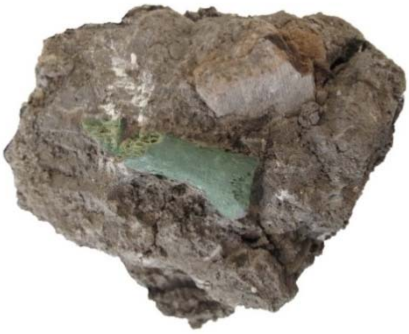

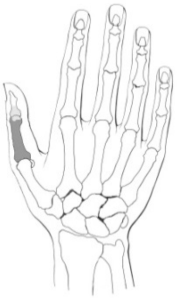

A single proximal and distal phalanx of the right first digit, lying on the palmar side.

L1777

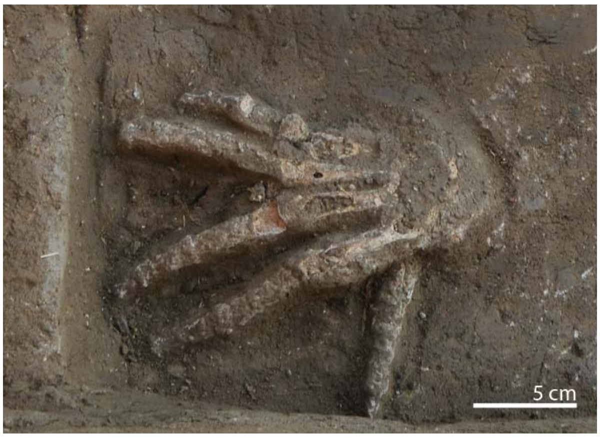

In situ

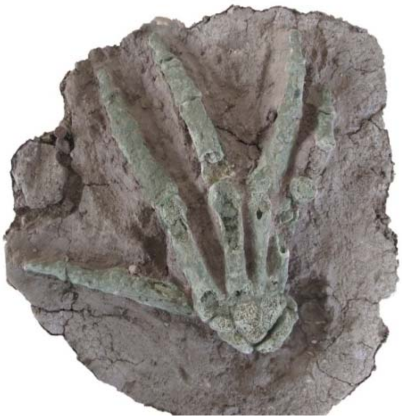

in lab

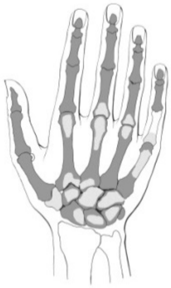

A single right hand placed on its palmar side. Preservation is quite poor with the surfaces eroded and covered by a hardened layer of calcareous deposits. The dorsal surface of the carpals and metacarpals were destroyed post-mortem. The hand ends with the carpals; no evidence of lower arm bones. The carpals cannot be assessed for cut marks due to their eroded surface. The fingers are spread with the largest distance between the third and fourth digits. Not fitting to this position is the first digit, which is also abducted, but its proximal part with the carpals and proximal half of the metacarpal placed below the hand.
